# Breast Cancer and Beyond: Screening, Psychosocial, and Socioeconomic Dimensions of Patient Experience During Radiotherapy in a United Arab Emirates Cohort

**DOI:** 10.7759/cureus.109681

**Published:** 2026-05-26

**Authors:** Chippy Radhakrishnan, Theresa Binz, Anusha Karakunnummal, Ruwayda Mokdad, Omama Mustafa Abdelrahman Salih, Manjula Nallappa, Nandan M Shanbhag, Abdulrahman Bin Sumaida, Khalid Balaraj

**Affiliations:** 1 Radiation Oncology, SEHA Tawam Hospital, Al Ain, ARE; 2 Radiation Oncology, University of Pittsburgh Medical Center (UPMC) Hillman Cancer Centre, Cork, IRL; 3 Oncology, SEHA Tawam Hospital, Al Ain, ARE

**Keywords:** body image, breast cancer, financial burden, psychosocial distress, radiotherapy, screening, supportive care, united arab emirates

## Abstract

Background

Breast cancer care extends beyond tumor-directed treatment to include screening behavior, psychosocial well-being, body image, family support, and financial and logistical burden. These issues are particularly relevant during radiotherapy, where repeated hospital attendance may amplify practical and emotional stressors. This study described multidimensional patient-experience domains among women receiving breast cancer care through the radiotherapy pathway at a tertiary cancer center in the United Arab Emirates.

Methods

A descriptive cross-sectional survey was conducted among 30 women with breast cancer managed through the radiation oncology pathway at SEHA Tawam Hospital, Al Ain, UAE, between August 12 and October 8, 2024. A structured bilingual questionnaire in Arabic and English was administered by tablet or QR code. Variables included demographic characteristics, screening participation, cancer detection pathway, lifestyle factors, financial concerns, perceived stress before and after diagnosis, body image, family support, and interest in supportive-care interventions. Descriptive statistics were used.

Results

The mean age at diagnosis among respondents with numeric age data was 47.4 years (SD 11.9; range 27-70; n=29). Most questionnaires were completed in Arabic (24/30, 80.0%) and by tablet (27/30, 90.0%). Most participants were married (17/30, 56.7%), 13 (43.3%) resided in Al Ain, and 20 (66.7%) were unemployed. Only 12 participants (40.0%) reported prior participation in a breast screening program, while 21 (70.0%) reported self-detection of a breast lump as the detection pathway. Physical activity of less than 30 minutes per day was reported by 17 participants (56.7%). Mean perceived stress increased from 5.3/10 before diagnosis to 6.9/10 after diagnosis. Mean scores also indicated high family/friend support (8.3/10), body image impact (6.0/10), support-group interest (5.4/10), financial impact (5.0/10), financial worry (5.2/10), and medical expense concern (4.7/10).

Conclusions

In this small single-center UAE cohort, women receiving breast cancer care through the radiotherapy pathway reported low screening participation, frequent self-detection, increased stress after diagnosis, body-image concerns, financial/logistical burdens, and interest in supportive-care interventions. These findings support integrating structured psychosocial assessment, screening outreach, and supportive-care pathways into breast radiotherapy services.

## Introduction

Breast cancer remains one of the most frequently diagnosed cancers worldwide and represents a major source of cancer-related morbidity and mortality among women [1]. In the UAE, breast cancer is the most common malignancy among women, and local data suggest a younger age at diagnosis and a substantial proportion of advanced presentations compared with many Western populations [2]. This UAE-based breast cancer cohort study included 372 participants, reported a median age at diagnosis of 48 years, with 24% of patients aged 40 years or younger at diagnosis. The same study found that only 12.3% of tumors were screen-detected, while 30% of patients presented with locally advanced disease and 20% with stage IV disease at presentation [2]. These observations make early detection, patient education, and patient-centered supportive care particularly relevant for UAE oncology services.

Screening participation remains a persistent challenge in the UAE. Recent and regional studies have identified gaps in mammography awareness, risk perception, and screening utilization among women in the UAE and Northern Emirates [3,4]. When breast cancer is detected through symptoms rather than organized screening, patients may enter oncology pathways with higher emotional burden, uncertainty, and practical challenges. These issues may be magnified during radiotherapy, where patients often attend treatment repeatedly over several weeks while managing post-surgical recovery, systemic therapy effects, work and family responsibilities, transport needs, and financial concerns.

Patient experience during breast cancer care is therefore shaped by more than tumor biology and treatment technique. Psychological distress is common among patients with cancer [5], body-image concerns are well described among breast cancer survivors [6], and financial burden can affect well-being and care decisions even when patients have some form of health coverage [7,8]. Supportive-care needs among breast cancer survivors include psychological, informational, physical, interpersonal, and practical domains [9]. Radiotherapy departments, because of their repeated contact with patients, may be well positioned to identify these needs and connect patients with counseling, education, rehabilitation, peer support, and social services.

This study aimed to describe the demographic, screening, lifestyle, psychosocial, body-image, financial, and supportive-care profile of women receiving breast cancer-related care through the radiotherapy pathway at SEHA Tawam Hospital, UAE. The objective was descriptive and service-improvement oriented: to identify patient-experience domains that may inform a more holistic breast radiotherapy pathway.

## Materials and methods

Study design and setting

This was a descriptive cross-sectional survey conducted at SEHA Tawam Hospital, Al Ain, Abu Dhabi, United Arab Emirates. The study followed a Strengthening the Reporting of Observational Studies in Epidemiology (STROBE)-informed approach for reporting observational cross-sectional data [10]. The survey was conducted among women receiving breast cancer care through the radiation oncology pathway.

Participants

Thirty women with breast cancer completed the survey. Participants were women undergoing assessment or treatment through the radiotherapy service who were able to complete the questionnaire in Arabic or English. The dataset contained 30 complete survey records; additional index-only rows in the spreadsheet were excluded from analysis.

Survey instrument and data collection

A structured bilingual questionnaire was developed in Arabic and English and administered electronically by tablet or QR code (Appendix 1). The questionnaire included items on demographics, residence, living situation, education, marital status, employment, financial concerns, family history, screening participation, method of cancer detection, physical activity, smoking and alcohol use, body image, intimate relationship impact, family/friend support, emotional well-being assessment, social/support-group interest, and perceived stress before and after diagnosis. Data collection occurred from August 12, 2024, to October 8, 2024.

Variables

The main variables included age at diagnosis, questionnaire language, survey mode, residence, living situation, marital status, education, employment status, site of diagnosis, self-reported stage, screening participation, detection method, physical activity, smoking status, alcohol use, accommodation and transport during radiotherapy, family/friend support, body-image impact, intimate relationship impact, financial impact, financial worry, medical expense concern, interest in interacting with other women undergoing treatment, support-group interest, and perceived stress before and after diagnosis.

Statistical analysis

Descriptive statistics were used. Categorical variables are reported as frequencies and percentages using non-missing denominators. Continuous and 0-10 scale variables are reported as mean, standard deviation, median, range, and missing values. Age at diagnosis was analyzed using numeric responses only. One participant entered a nonnumeric response ("before 2 years") and was excluded from the primary age calculation. Formal normality testing was not performed, as the analysis was descriptive and no inferential parametric testing was planned. No inferential testing was performed because the study was descriptive and the sample size was small. Descriptive analyses were performed using spreadsheet-based calculations and independently recomputed from the final Excel dataset (Microsoft, Redmond, WA, USA).

Ethical considerations

Consent was obtained or waived by all participants in this study. Tawam Human Ethics Committee/Al Ain Region Human Research Ethics Committee issued approval MF2058-2025-1240.

## Results

Participant characteristics and survey workflow

Thirty women completed the survey. Numeric age-at-diagnosis data were available for 29 participants; one participant had missing/non-numeric age data and was therefore excluded from age-summary calculations. The median age at diagnosis was 44.0 years, with a range of 27-70 years. The mean age was 47.4 years, with a relatively wide standard deviation of 11.9 years, reflecting variability within this small cohort.

Most surveys were completed in Arabic (24/30, 80.0%) and by tablet (27/30, 90.0%). Seventeen participants (56.7%) were married, 13 (43.3%) had a bachelor’s degree or above, and 13 (43.3%) resided in Al Ain. Twelve participants (40.0%) were diagnosed at Tawam Hospital, while 18 (60.0%) were diagnosed elsewhere and referred for care. Self-reported stage information was heterogeneous; stage 2 was the most common reported stage among non-missing responses (11/29, 37.9%), followed by stage 3 (8/29, 27.6%) (Table 1).

**Table 1:** Participant demographic and background characteristics

Characteristic	Value
Sample size	30
Survey period	August 12, 2024 to October 08, 2024
Age at diagnosis, years, mean ± SD	47.4 ± 11.9 (n=29)
Age at diagnosis, years, median (range)	44.0 (27-70)
Language	
Arabic	24 (80.0)
English	6 (20.0)
Survey mode	
Tablet	27 (90.0)
QR code	3 (10.0)
Residence	
Al Ain	13 (43.3)
Dubai	5 (16.7)
Ajman	4 (13.3)
Sharjah	3 (10.0)
Abu Dhabi	2 (6.7)
Umm Al Quwain	2 (6.7)
Ras Al Khaimah	1 (3.3)
Living situation	
Lives with others	27 (90.0)
Lives alone	3 (10.0)
Diagnosed at	
Tawam Hospital	12 (40.0)
Other hospital	18 (60.0)
Marital status	
Married	17 (56.7)
Single	5 (16.7)
Divorced	5 (16.7)
Widowed	3 (10.0)
Education	
No formal schooling	3 (10.0)
Primary	2 (6.7)
Secondary	6 (20.0)
Diploma	6 (20.0)
Bachelor or above	13 (43.3)
Reported stage	
Stage 0	2 (6.9)
Stage 1	2 (6.9)
Stage 2	11 (37.9)
Stage 3	8 (27.6)
Advanced/unspecified	1 (3.4)
Don't know	1 (3.4)
Other/unclear	4 (13.8)

Socio-economic, logistical, and family-support characteristics

Twenty participants (66.7%) were unemployed. Fourteen (46.7%) planned to stay in Tawam-provided accommodation during radiotherapy, 13 (43.3%) reported less than one hour of daily travel, and three (10.0%) reported more than one hour of daily travel. Eighteen participants (60.0%) reported that a family member or friend would accompany them daily for radiotherapy, while four (13.3%) planned to attend alone. Most participants reported that family and friends knew about the diagnosis (27/30, 90.0%). Family history of breast cancer was reported by eight participants (26.7%) (Table 2).

**Table 2:** Socio-economic, logistical, and family-support characteristics

Characteristic	n (%) or value
Employment status	
Employed	10 (33.3)
Unemployed	20 (66.7)
Accommodation during radiotherapy	
Tawam accommodation	14 (46.7)
<1 hour daily travel	13 (43.3)
>1 hour daily travel	3 (10.0)
Transport mode	
Own vehicle	10 (33.3)
Hospital accommodation	12 (40.0)
Driver	4 (13.3)
Taxi	3 (10.0)
Bus	1 (3.3)
Accompaniment to radiotherapy	
Family/friend daily	18 (60.0)
Varies	8 (26.7)
Comes alone	4 (13.3)
Family/friends aware of diagnosis	
Yes	27 (90.0)
No	3 (10.0)
Family history of breast cancer	
Yes	8 (26.7)
No	22 (73.3)

Screening, detection, lifestyle, and supportive-care variables

**Figure 1:**
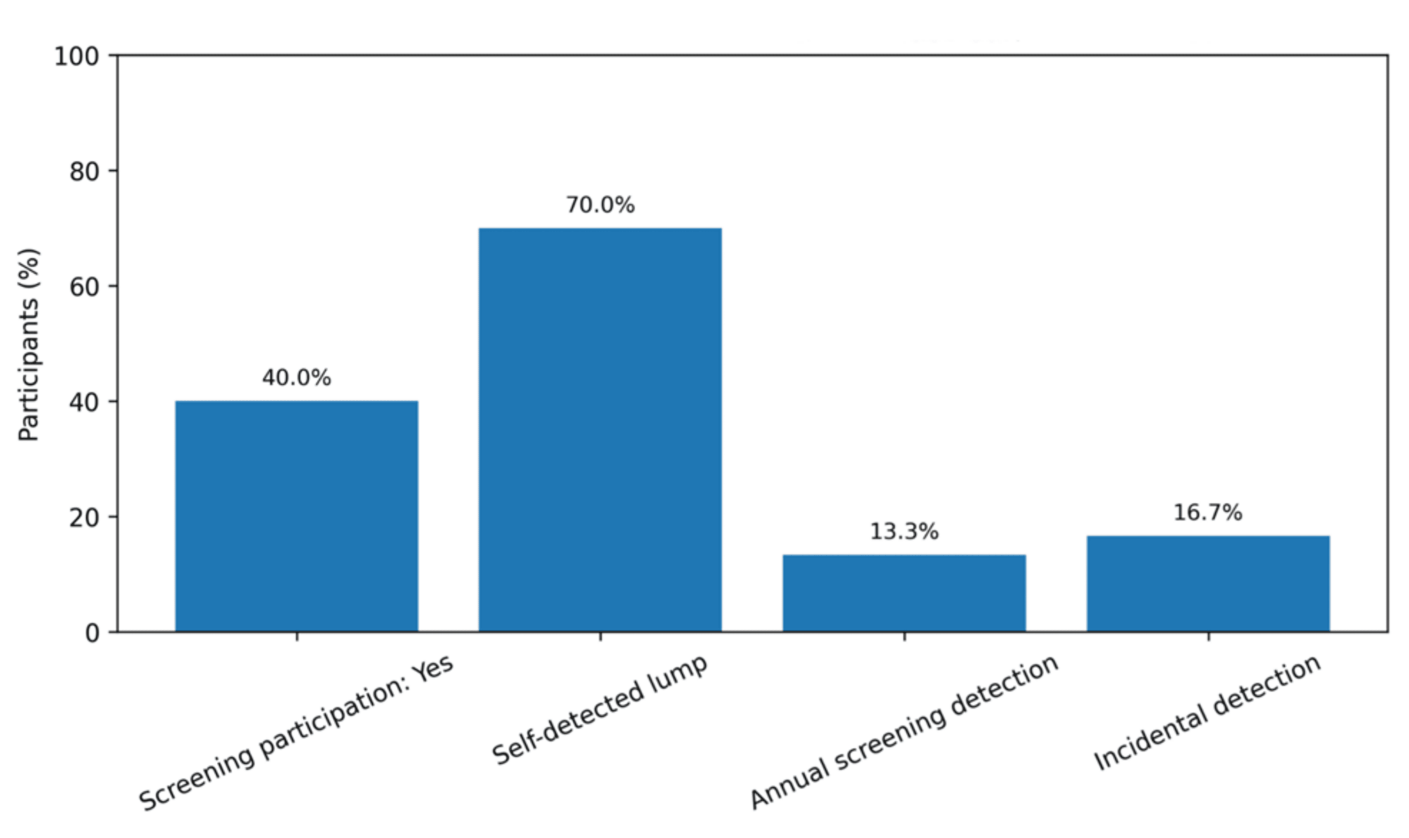
Screening participation and method of breast cancer detection. Percentages were calculated using N=30.

Only 12 participants (40.0%) reported prior participation in a breast screening program. Breast cancer was detected through self-detection of a breast lump in 21 participants (70.0%), after annual screening in four (13.3%), and incidentally in five (16.7%). Physical activity of less than 30 minutes per day was reported by 17 participants (56.7%). Twenty-nine participants (96.7%) reported not smoking, and all 30 reported no alcohol consumption. Only three participants (10.0%) reported ongoing physiotherapy after surgery, while 27 (90.0%) reported no physiotherapy (Table 3, Figure 1).

**Table 3:** Screening, detection, lifestyle, and supportive-care variables

Characteristic	n (%) or value
Screening participation	
Yes	12 (40.0)
No	18 (60.0)
Detection method	
Self-detected breast lump	21 (70.0)
Detected after annual screening	4 (13.3)
Incidental finding	5 (16.7)
Physical activity	
<30 min/day	17 (56.7)
30 min-1 hour/day	6 (20.0)
>1 hour/day	7 (23.3)
Smoking status	
No	29 (96.7)
Cigarettes	1 (3.3)
Alcohol consumption	
None	30 (100.0)
Pre-diagnosis breast lump history	
Yes	6 (20.0)
No	24 (80.0)
Post-surgery physiotherapy	
No	27 (90.0)
Still ongoing	3 (10.0)

Psychosocial, body-image, stress, and financial burden findings

**Figure 2:**
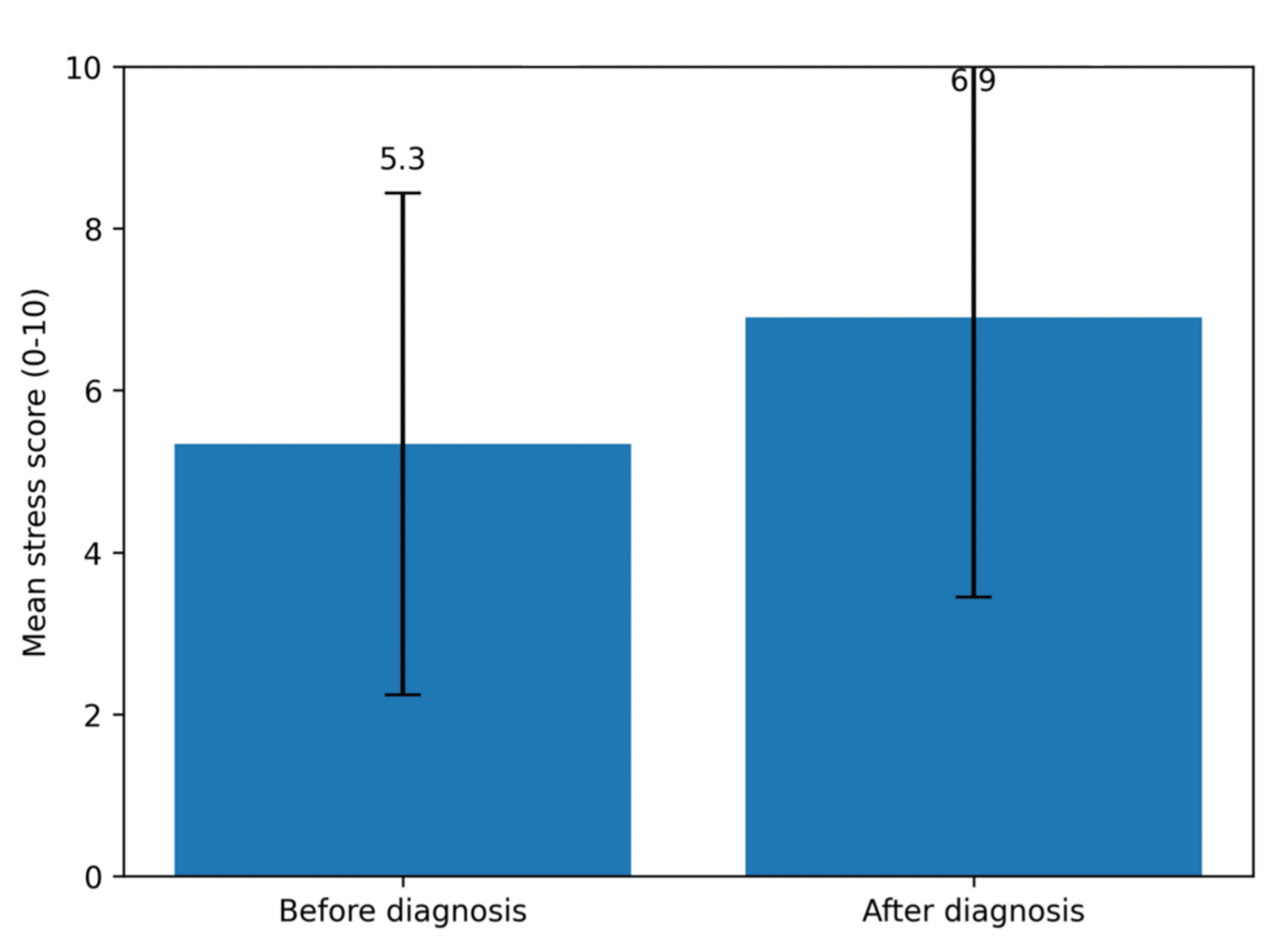
Mean perceived stress before and after breast cancer diagnosis. Error bars show standard deviation. Scores were reported on a 0-10 scale.

Mean perceived stress increased from 5.3/10 before diagnosis to 6.9/10 after diagnosis (Figure 2).

Mean family/friend support was high at 8.3/10, but several psychosocial and financial concerns were evident. Mean body-image impact was 6.0/10, mean emotional well-being assessment by the medical team was 6.0/10, mean support-group interest was 5.4/10, and mean interest in interacting socially with other women undergoing treatment was 6.5/10. Mean financial impact was 5.0/10, mean financial worry was 5.2/10, and mean medical expense concern was 4.7/10. Using a threshold of 5 or higher on 0-10 scales, 19 participants (63.3%) reported at least moderate negative financial impact, 19 (63.3%) reported at least moderate financial worry, 21 (70.0%) reported at least moderate body-image impact, and 20 (66.7%) reported at least moderate interest in joining a support group during radiotherapy (Table 4).

**Table 4:** Psychosocial, body-image, stress, and financial burden scores Values are n (%) unless otherwise stated. Percentages were calculated using non-missing denominators. Numeric scores are based on 0-10 response scales unless otherwise stated. Age at diagnosis uses numeric responses only.

0-10 scale variable	n	Mean ± SD	Median (range)	Missing, n
Financial impact of diagnosis	30	5.0 ± 4.0	5.0 (0-10)	0
Financial worry	30	5.2 ± 4.1	5.0 (0-10)	0
Medical expense concern	30	4.7 ± 4.4	5.0 (0-10)	0
Intimate relationship impact	24	3.4 ± 4.2	0.0 (0-10)	6
Body image impact	30	6.0 ± 3.7	7.5 (0-10)	0
Interaction with fellow patients helpful	30	6.5 ± 3.6	7.0 (0-10)	0
Weekly social gathering interest	30	4.1 ± 4.0	5.0 (0-10)	0
Support group interest	30	5.4 ± 4.0	5.5 (0-10)	0
Family/friend support sufficient	30	8.3 ± 2.6	10.0 (2-10)	0
Emotional well-being regularly assessed	30	6.0 ± 4.0	7.0 (0-10)	0
Perceived control of life	29	7.4 ± 2.5	8.0 (2-10)	1
Life changed by diagnosis	30	6.9 ± 3.5	8.0 (0-10)	0
Family distress	30	7.5 ± 2.8	8.5 (0-10)	0
Concern for daughter/female relatives	30	5.0 ± 4.3	5.0 (0-10)	0
Stress before diagnosis	30	5.3 ± 3.1	5.5 (0-10)	0
Stress after diagnosis	30	6.9 ± 3.5	8.0 (0-10)	0

## Discussion

This descriptive cross-sectional survey highlights several patient-experience domains among women receiving breast cancer care through the radiotherapy pathway in a UAE tertiary cancer center. The main findings were low prior screening participation, predominance of self-detected disease, frequent unemployment, meaningful financial and logistical concerns, increased stress after diagnosis, body-image impact, and interest in structured supportive-care activities. Although family support was high, this did not eliminate emotional, financial, or body-image concerns.

The screening and detection findings are clinically important but should be interpreted cautiously given the small sample size and the inclusion of participants below the usual screening-eligible age group. In this cohort, 40.0% of participants reported prior participation in a breast screening program, whereas 70.0% reported self-detection of a breast lump. While these findings cannot establish a population-level gap in screening uptake, they are consistent with previously published UAE literature describing suboptimal screening awareness and utilization [3,4]. They also align with UAE clinical data showing that screening-detected tumors remain a minority of presentations in some cohorts [2]. The radiotherapy pathway may therefore offer an opportunity not only to deliver treatment but also to reinforce age-appropriate, family-centered screening education, especially for daughters and female relatives of affected women.

The psychosocial findings support the need for structured distress assessment during breast radiotherapy. Mean stress increased after diagnosis, and body-image impact was notable. Psychological distress is common in patients with cancer [6], and body-image concerns after breast cancer diagnosis and treatment are well recognized, particularly among younger women and those affected by surgery, systemic therapy, and changes in sexuality or intimacy [7]. In this cohort, the mean emotional well-being assessment score was moderate, suggesting that patients may not consistently perceive psychosocial review as part of routine care.

Financial and logistical burdens were also evident. Two-thirds of participants were unemployed, almost half required Tawam-provided accommodation, and 10.0% reported more than one hour of daily travel for treatment. Mean financial impact, financial worry, and medical expense concern scores were moderate. Financial toxicity has been described as a multidimensional cancer-care burden affecting material resources, psychological well-being, and treatment behavior [8]. Breast cancer-specific studies similarly show that patients may experience distress from medical and nonmedical costs, missed work, and altered budgeting [9]. In the UAE setting, even when direct treatment costs are covered or subsidized, indirect costs such as travel, accommodation, caregiving, time away from work, and family disruption may remain important.

The interest in supportive-care interventions is actionable. Two-thirds of participants reported at least moderate interest in joining a support group, and more than 80% reported that interacting with fellow female patients would be helpful. Systematic reviews of supportive-care needs among breast cancer survivors highlight psychological, informational, physical, interpersonal, and practical needs [10]. Evidence for psychosocial rehabilitation interventions is mixed but suggests potential quality-of-life benefit for selected interventions such as cognitive behavioral therapy after primary treatment [11]. Integrating routine distress screening, counseling pathways, physiotherapy referral, peer-support options, and survivorship education may therefore improve the patient experience during and after radiotherapy [12].

This study has practical implications for radiotherapy services. Repeated treatment visits create multiple points of contact during which distress, financial concerns, transport barriers, family needs, physical activity, body image, and rehabilitation gaps can be identified. A structured breast radiotherapy supportive-care checklist could include screening history, family education needs, distress score, body-image concerns, financial/logistical concerns, physiotherapy status, and interest in peer support. Such an approach may help move breast cancer care beyond treatment delivery alone toward a more holistic patient-centered pathway.

Strengths and limitations

A strength of this study is its focus on domains often underreported in routine oncology datasets, including screening behavior, financial concern, stress, body image, family support, and support-group interest. The bilingual electronic questionnaire improved accessibility for Arabic- and English-speaking patients.

Limitations include the small sample size, single-center design, and descriptive cross-sectional methodology. The findings are not intended to establish causality or generalize to all breast cancer patients in the UAE. Survey responses were self-reported and may be affected by recall bias, social desirability bias, and response interpretation. Surgical modality, including mastectomy versus breast-conserving surgery, was not consistently captured in the survey dataset. This limits interpretation of body-image findings, as surgical approach may significantly influence body image, intimacy, and psychosocial adjustment. The questionnaire was locally developed and was not documented as a validated psychometric instrument. Several variables, including self-reported stage and age at diagnosis, included some ambiguous entries. No inferential statistical testing was performed. These findings should therefore be interpreted as hypothesis-generating and service-improvement oriented.

## Conclusions

Women receiving breast cancer care through the radiotherapy pathway at Tawam Hospital reported multidimensional needs extending beyond tumor-directed treatment. Low screening participation, frequent self-detection, increased stress after diagnosis, body-image impact, financial/logistical concerns, and interest in supportive-care interventions were evident in this small UAE cohort. Radiotherapy departments may be well placed to integrate structured psychosocial assessment, screening education, rehabilitation referral, and peer-support pathways into routine breast cancer care.

## Appendices

Appendix 1: Google survey questionnaires link

https://docs.google.com/forms/d/1zesfa1IBVzDTcA0RQRVw_7rUynLkGkw3bsUWLGCpgYE/edit?pli=1
